# Semiautomated Monitoring of Longitudinal Microbial Metabolic Dynamics: A Study Case for Lignin Degradation

**DOI:** 10.1021/acsomega.5c05607

**Published:** 2026-01-26

**Authors:** João Vítor Guimarães Ferreira, Gabriel Santos Arini, Tiago Cabral Borelli, Isabela Victorino da Silva Amatto, Winner Duque Rodrigues, Igor Sepulveda Rodrigues, Nathália Gonsales da Rosa-Garzon, Izabel Cristina Casanova Turatti, Henrique Marcel Yudi de Oliveira Tsuji, Iasmin Cartaxo Taveira, Livia Soares Zaramela, Norberto Peporine Lopes, Hamilton Cabral, Ricardo Roberto da Silva

**Affiliations:** † School of Pharmaceutical Sciences of Ribeirão Preto, 67782 University of São Paulo, Ribeirão Preto, São Paulo 14040-900, Brazil; ‡ Faculty of Philosophy, Sciences and Letters at Ribeirão Preto, University of São Paulo, Ribeirão Preto, São Paulo 14040-900, Brazil; § Ribeirão Preto Medical School, 28133 University of São Paulo, Ribeirão Preto, São Paulo 14040-900, Brazil

## Abstract

This study introduces a low-cost, open-source, semiautomated bioreactor system, developed using Arduino and Raspberry Pi, for longitudinal microbial culture studies. The system protocol includes detailed instructions for custom-designed components, specifications for easily purchasable parts, and open-source code for a web-based control interface. It was validated for sterility and tested in a case study involving the cultivation of *Phanerochaete chrysosporium* and *Trichoderma reesei* to assess their metabolic profile, both in isolation and coculture, for lignin degradation. Using mass spectrometry coupled with gas chromatography, several lignin degradation intermediates were annotated, including 4-hydroxybenzoic acid, vanillic acid, and ferulic acid, along with their temporal detection over 25 days. Additionally, we developed an annotation workflow to search for enzymatic functions producing these compounds in the genomes of *P. chrysosporium* and *T. reesei*, providing multiple layers of evidence to describe, for the first time, a computational annotation for lignin metabolism in these fungi. The results highlighted the production of lignin breakdown intermediates by the two fungal species, with annotations of the respective enzymatic functions, while also demonstrating the bioreactor’s flexibility and suitability for diverse biotechnological applications, particularly in the field of biodegradation and waste valorization.

## Graphical abstract

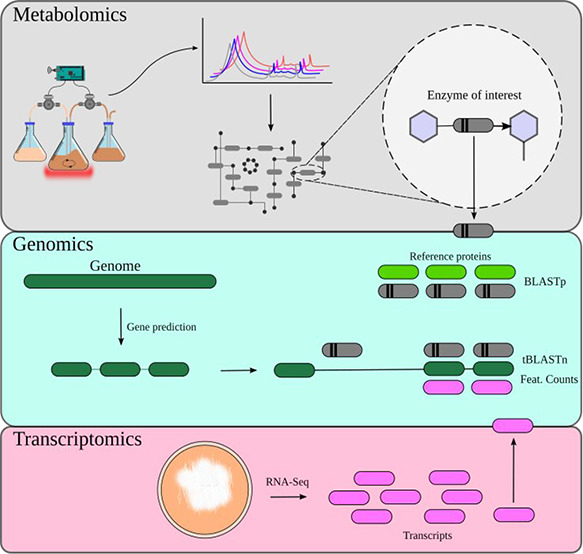

## Introduction

Fundamental to the carbon recycling dynamics within terrestrial ecosystems, lignin depolymerization represents not only an important technical challenge but also a focal point of interest for industries such as biorefinery.^1^ This interest stems from the potential to derive high-value products from this intricate natural polymer, notably low molecular weight aromatic chemicals that hold promise as substitutes for petroleum derivatives.^2^

Lignin, known for being a naturally occurring amorphous polymer, constitutes an essential component of lignocellulosic biomass, typically comprising 15–30% of such matrices.^3^ Its complex, highly branched, three-dimensional phenolic structure comprises three primary monolignols: *p*-coumaryl alcohol, coniferyl alcohol, and sinapyl alcohol;^4^ while its molecular mass exhibits great variability—a consequence of the random cross-linking facilitated by phenolic group polymerization.^4^ Such polymerization involves the coupling of monolignol units via peroxidase-mediated dehydrogenation, which results in the formation of C–C bonds and aryl ether linkages, including aryl-glycerol and β-aryl ether connections.^4^

Due to this intricate structure, lignin is highly resistant to degradation, presenting significant hurdles to its efficient utilization as a biomass resource within the industry.^5^ Traditionally, lignin is degraded in the pulp and paper industry using compounds such as chlorine dioxide, which are not environmentally friendly and pose additional concerns.^6^ In response to this challenge, the use of biodegradation has emerged as a prominent solution, offering numerous advantages, such as environmental friendliness, low energy consumption, and mild reaction condition requirements.^7^ Among the microorganisms capable of this process, white-rot fungi stand out as one of the most efficient actors, particularly due to their unique ligninolytic enzymes.^8^ These enzymes possess peculiar properties, including mediator utilization and surface-active sites, which enhance their redox potential and contribute to their effectiveness in lignin degradation.^9^ Remarkably, *Phanerochaete chrysosporium*, the most studied of all white-rot fungi, exemplifies this phenomenon through its extracellular enzymes (e.g., lignin peroxidase, manganese peroxidase, laccase), which efficiently depolymerize lignin.^10,11^

To date, several studies have been conducted using *P. chrysosporium* and its enzymes to degrade lignin and assess compositional changes in the resultant products at the microscale. For this purpose, scanning electron microscopy,^12^ Fourier-transform infrared spectroscopy,^13^ and gas chromatography coupled to mass spectrometry^14^ have been commonly employed methodological approaches. However, these studies typically focus on identifying these products for a single time point, corresponding to a single short period of culture, failing to capture the temporal dynamics of the fungus’ behavior during the degradation process. Additionally, there is a notable absence of comprehensive genomic annotation for the enzymes involved in lignin degradation in this fungal species, which limits a deeper understanding of their functional roles and regulatory mechanisms.^15^ Addressing this gap is essential, as exploring such a perspective could provide valuable insights into the changes over time in the fungus’ metabolic activities and their consequences for lignin degradation.

Another approach that has been increasingly employed to enhance the evaluation of the ligninolytic capabilities of white-rot fungi is the development of cocultures.^16^ These cocultures aim to achieve synergistic effects by combining different metabolic pathways or by inducing the expression of normally latent pathways, ultimately leading to greater efficiency in lignin degradation processes.^17^ One species identified as a potential contributor in coculture with white-rot fungi is *Trichoderma reesei*, a filamentous fungus widely studied for its ability to produce cellulose and lignin-degrading enzymes.^18,19^ Unlike *P. chrysosporium* and many other fungal species, *T. reesei* benefits from extensive publicly available genomic resources, including well-annotated genomes, which facilitate detailed mechanistic studies^20^ and make it stand out as an excellent candidate for coculture studies.^21^

To enable cocultures over time, we introduced an automated Arduino-based bioreactor system that maintains a controlled environment and allows for periodic sampling to analyze metabolite production. The developed bioreactor system was designed to be simple, cost-effective, and easy to assemble, making it accessible to users without technical skills in electronics or programming. Its components are readily available, and its programming is user-friendly, even for those with limited experience. This was made possible by utilizing the Arduino platform, an inexpensive, straightforward, and highly versatile microcontroller. In recent years, Arduino has become increasingly popular for laboratory applications, both in educational settings^22^ and research environments.^23^ Its main advantage lies in its versatility and ease of use, enabling the creation of customized systems for a wide range of applications.

By combining these two approaches—the temporal analysis of microbial behavior in lignin degradation and the evaluation of species interactions in coculture—this study aimed to provide new insights into lignin degradation. We monitored a coculture of *P. chrysosporium* and *T. reesei* over a 25-day period to assess the dynamics of both their metabolite production and consumption through GC-MS analysis. This longitudinal study allowed us to observe growth variation patterns in key compounds involved in lignin degradation, such as 4-hydroxybenzoic acid, vanillic acid and ferulic acid.

Given the absence of consensus metabolic pathways for lignin degradation in the two promising species for biotechnological applications, we investigated the presence of enzymatic functions that would produce the observed metabolites in the genomes of *P. chrysosporium* and *T. reesei*, and in the transcriptome of *P. chrysosporium*. We developed an annotation workflow to search for a given enzymatic function, providing multiple layers of evidence, including motif sequence similarity, transcriptional evidence, and structural similarity to describe, for the first time, a computational annotation for lignin metabolism in *P. chrysosporium* and *T. reesei*.

Additionally, the detailed documentation to reproduce a low-cost bioreactor and a full bioinformatics workflow to detect temporal metabolic transformations and annotate enzymatic functions has the potential to expand the knowledge repertoire in the temporal dynamics of lignin degradation.

## Materials and Methods

### Development of the Bioreactor

The bioreactor was built using an Arduino UNO, following the circuit design described here (https://github.com/computational-chemical-biology/arduino-based-bioreactor). The transfers were executed using peristaltic pumps (Intllab, flow rate of 450 mL/min) with an activation duration of 15 s, experimentally determined based on the density of the culture medium and the tubing size so that 50 mL of medium would be transferred in each activation. A Raspberry Pi 1 B+ board was used to create a web server controlling the Arduino board, following the application design described here (https://github.com/computational-chemical-biology/arduino-based-bioreactor/tree/main/flask app). To improve the system, we also designed custom Teflon caps and stainless-steel cannulas at the university’s precision workshop (https://www.prefeiturarp.usp.br/page.asp?url=precisao). The description of these parts with exact dimensions is included in the project’s GitHub page.

### System Validation

System validation occurred in two stages. First, a test was conducted to verify the maintenance of sterility inside the system. For this, LB medium was used in two of the glassware containers (feeding and culturing), with 200 and 100 mL, respectively. The set of three glassware containers and their corresponding tubing was autoclaved for 15 min at 121 °C. Subsequently, the system was assembled and put into operation, with the contents of the central flask (where cultivation would take place) being transferred to the collection flask, and then from the feeding flask to the cultivation flask every 5 days. Three complete cycles were performed. The material from the collection flask was collected every 5 days and discarded. At the end of the third cycle, the contents of the three flasks were plated on Petri dishes with PDA medium and incubated at 30 °C for 7 days. Second, a monoculture of *P. chrysosporium* was carried out in a lignin-containing culture medium, with five-day cycles for a period of one month. After completion, the contents of the containers were plated on PDA medium, following the same protocol as before.

### Culture Conditions

The inoculation of the cultivation unit involved using 20 discs, each 1 cm in diameter, for *P. chrysosporium*, and a spore suspension of *T. reesei* (QM 9414 M) at a concentration of 5.0 × 10^6^ spores/ml. Both species were previously cultured for 6 days at 30 °C in Petri dishes before their final inoculation into the system. The experiments were conducted in 5-day cycles over a period of 25 days. Each cycle began with the transfer of 50 mL from the culture flask to the collection one, followed by the transfer of 50 mL from the feeding unit to the culture one. The culture container was subjected to agitation and heating using an IKA C-MAG HS7 magnetic stirrer (agitation speed level 2 (500 rpm), heating at 30 °C) and aeration by an aquarium air compressor (Maxxi Pro-5000). The temperature was controlled by a magnetic stirrer, which was located in the same room with air conditioning set to 22 °C. The culture medium composition was as follows: 0.7% w/v KH_2_PO_4_, 0.2% w/v K_2_HPO_4_, 0.01% w/v MgSO_4_·7H_2_0, 0.05% w/v Sodium citrate (dihydrate), 0.1% w/v Yeast extract, 0.01% w/v CaCl_2_·2H_2_O, 0.1% w/v Peptone, and 0.5% w/v Lignin (kraft lignin, kindly donated by Klabin - https://klabin.com.br/), with a pH of 6.0. At the beginning, the feeding and cultivation units contained 400 and 100 mL of culture medium, respectively, while the collection container was empty. Throughout the experiment, the volume of the culture flask remained constant.

### Collection Protocol

To ensure the maintenance of sterility within the system, samples were periodically collected from the collection flask, which was equipped with an outlet tubing sealed by a surgical clamp. On sampling days, the outlet tubing was sanitized with 70% v/v ethanol before the surgical clamp was removed, and the contents were extracted using a sterile 60 mL syringe. The sample was then transferred to 50 mL Falcon tubes and frozen at −80 °C.

### Validation Experiments

As the current version of our bioreactor is only capable of performing a single cultivation for time series, a validation experiment was necessary to confirm the trends observed in this prototype. In order to verify the metabolite fluctuation over time and substantiate biological meaning, we performed cultures of *P. chrysosporium*, *T. reesei*, coculture, and a control, composed of sterile Lignin media, as described above, with three biological replicates in a shaker (New Brunswick I26 Incubator Shaker) at 120 rpm and 30 °C over 25 days. The inoculum for each cultivation was made at the same concentrations described above. The cultures were conducted in 5-day cycles over a period of 25 days. In each 5-day cycle, 25 mL of the culture/control was removed and stored at −80 °C and 25 mL of culture/control was reinoculated into 25 mL Erlenmeyer flasks of fresh medium. Additionally, a growth curve for the fungi *P. chrysosporium*, *T. reesei*, and the coculture was performed using an inoculum of 5 × 10^6^ CFU/mL of each fungus in 30 mL of modified Czapek Dox medium (Sucrose 30 g/L, NaNO_3_ 3 g/L, K_2_HPO_4_ 1 g/L, MgSO_4_·7H_2_O 0.5 g/L, KCl 0.5 g/L, FeSO_4_·7H_2_O 0.01 g/L), with the pH adjusted to 6.0. The experiment was performed in biological triplicate, with the negative control consisting of the culture medium without inoculum. The flasks were incubated at 30 or 22 °C with agitation at 180 rpm. Aliquots were withdrawn over time, and the OD_600_ was measured using a spectrophotometer.

### GC-MS Analysis

The analyses of the collected samples were carried out using gas chromatography coupled with mass spectrometry (GC-MS). The samples were thawed at room temperature and then centrifuged at 8,000 *g* for 10 min in order to separate the supernatant from the cellular fraction. The supernatant was collected, and the pH was adjusted to 2.0 using a 6 mol/L HCl solution. Liquid extraction was then performed with the addition of 2 mL of ethyl acetate in three separate steps. The organic phase was collected and dehydrated with Na_2_SO4. Once dehydrated, the sample was dried under a stream of nitrogen gas and derivatized. For derivatization, 20 μL of pyridine and 100 μL of bis­(trimethylsilyl)­trifluoroacetamide (BSTFA) were added. The solution was placed in a water bath at 60 °C for 30 min, with regular shaking. The resulting silylated samples were then injected into GC-MS QP2010 Ultra (Shimadzu, Kyoto, Japan). A ZB-5MS capillary column (30 m × 0.25 mm inner diameter × 0.25 μm film thickness) was used, with helium as the carrier gas at a flow rate of 1 mL/min. The column temperature started at 50 °C (held for 5 min) and was increased to 300 °C at a rate of 10 °C per minute. The injection temperature was 280 °C, with the ion source maintained at 250 °C and the transfer line at 200 °C. The solvent delay was set to 5 min. The injection volume was 1 μL. The acquisition of fragmentation spectra for the standards of ferulic acid (Sigma-Aldrich) and vanillic acid (Sigma-Aldrich) was performed under the same instrumental conditions.

### Data Processing

The Shimadzu QGD files were converted to an open format (.mzXML) with OpenChrom software (v. 15.0)^24^ and preprocessed through MZmine 2.53 ^25^ following the parameters presented in the .xml file available in the project’s GitHub page. The preprocessed data were subsequently annotated through spectral pairing through the GNPS platform,^26^ using the MOLECULAR-LIBRARY SEARCH-GC workflow. The full results of the spectral matching, as well as all the adopted parameters, are available at https://gnps.ucsd.edu/ProteoSAFe/status.jsp?task=cc5af8857436441f9373a6bda0a17199 (accessed on 7 July 2024).

### Metabolomics Temporal Correlation Analysis

To verify the correlation between the peak areas for each compound annotated at the evaluated time points (i.e., increase or decrease over time), an analysis was conducted using the Pearson correlation coefficient. A threshold value of 0.80 (in absolute terms) was established to filter correlations between the peak areas under study. The detailed calculations and analysis steps can be reviewed in the Jupyter Notebook available on the project’s GitHub page.

### Comparative Genomics to Search for Lignin-Degrading Enzymes

We investigated the presence of genes encoding lignin-degrading enzymes in the reference genomes of *P. chrysosporium* ATCC 20696 (GCA_001910725.1) and *T. reesei* QM6a (GCF_000167675.1). Unlike *T. reesei*, the *P. chrysosporium* genome lacks gene, protein and coding sequence annotations, therefore, we employed *de novo* gene prediction using Augustus 3.1.0 (Keller et al., 2011 ^200^). To search for genomic evidence of enzymes catalyzing the reactions described in Figure 2 and producing the metabolites detected in our GC-MS experiments, we retrieved curated enzyme sequences from Swiss-Prot based on their Enzyme Commission (EC) numbers and used them as queries in a standalone tBLASTn search (max_target_seqs = 5) against the *P. chrysosporium* and *T. reesei* genome sequences. The resulting aligned regions were then mapped to their respective gene loci. Protein sequences from these overlapping regions were extracted and validated using a standalone BLASTp search against Swiss-Prot enzymes for functional confirmation. Transcriptomic data from a public study (BioProject: PRJNA285669) were reanalyzed to validate expression of the genes identified above. SRA-toolkit 3.2.0 was used to download SRA runs and extract the FASTQ files. Quality control was performed using Trimmomatic 0.39 ^27^ with parameters: LEADING:3; TRAILING:3; SLIDINGWINDOW:4:15; MINLEN:36. Genome reference indexing and mapping was performed using STAR 2.7.11,^28^ and featureCounts 2.0.8 was used to count the number of reads mapped back to the Augustus-predicted coding sequences and report the reads per kilobase per million mapped reads (RPKM). Differential gene expression analysis was performed by PyDeseq2 0.5.0.^29^ The tool gffread 0.12.7 ^30^ was employed for intron removal and for translating coding sequences into protein sequences, which were aligned against Swiss-Prot enzymes using DIAMOND 2.1.11.165.^31^ The 3D structure analysis of target proteins was performed with AlphaFold for protein modeling and PyMOL for structure alignment.

## Results and Discussion

### Design and Development of the Bioreactor

A schematic of the system can be seen in Figure 1a–b. In simplified terms, the developed bioreactor system consists of three essential parts: (1) three 500-ml glass containers serving as the feeding, cultivation, and collection units of the system, equipped with custom-made Teflon caps with spaces for attaching stainless-steel cannulas through which the contents of the containers pass; (2) two peristaltic pumps, connected to the stainless-steel cannulas via silicone tubings, directing the transit of the contents; (3) a central control system, consisting of an Arduino UNO board, an integration circuit board, and a Raspberry Pi 1 B+ board, responsible for storing and executing the programmable commands that govern the overall functioning of the system. Additional elements that facilitated the operation of the experiments included magnetic stirrers with heating, an air compressor for aquariums, 0.22 μm membrane filters, and a surgical clamp. The magnetic stirrers maintained the culture temperature and ensured uniform distribution of nutrients in the culture medium. The air compressor aerated the cultivation medium, which was essential for the growth of the species studied. The membrane filters prevented pressure imbalances and system contamination during the experiments. Similarly, the surgical clamp prevented contamination by the outlet tubing, which was only opened during sample extraction, in accordance with a validated protocol.

**Figure 1:**
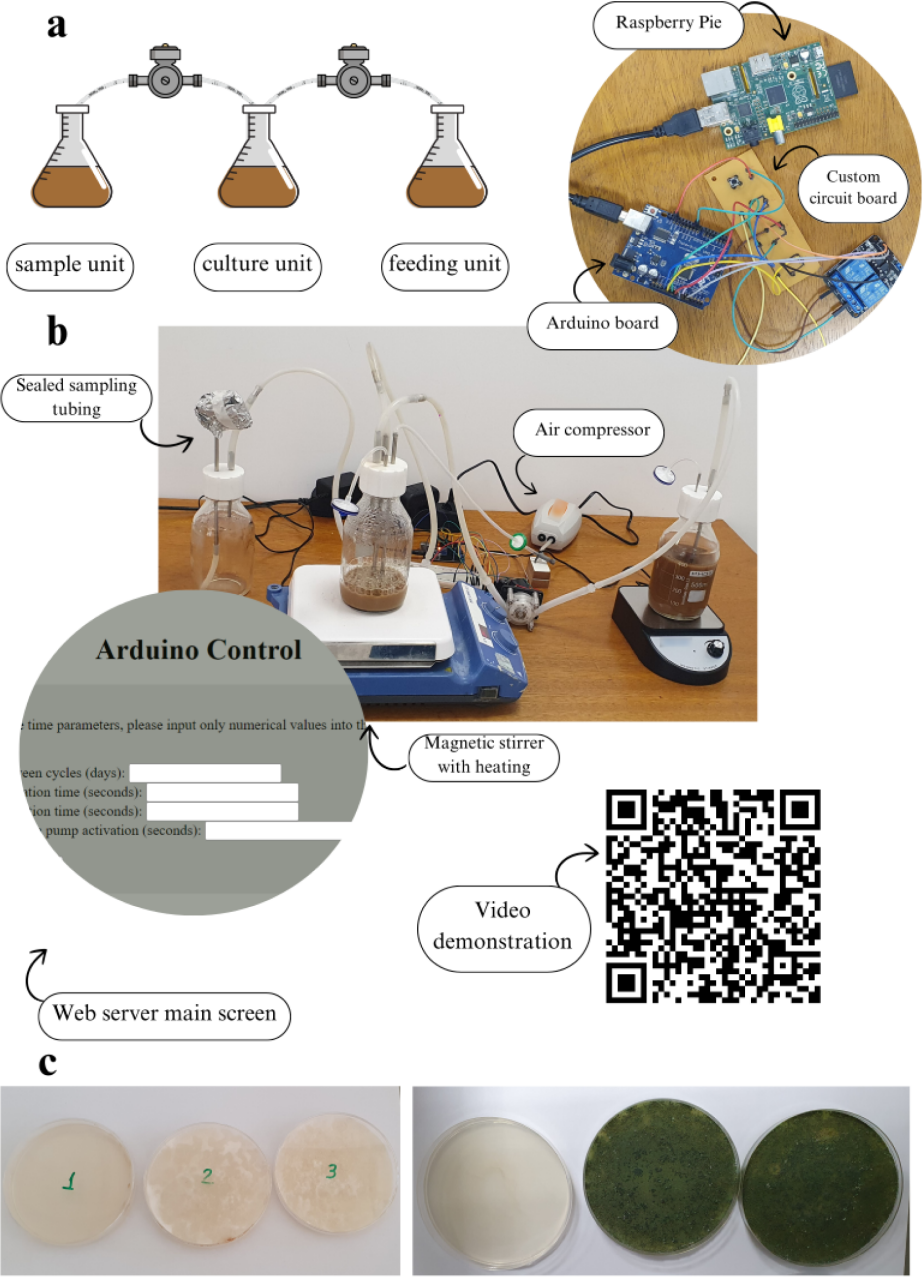
Overview of the bioreactor system and postcultivation contamination analysis. A) Schematic diagram of the bioreactor setup. B) Photograph of the operational system, highlighting key components, including the customized circuit, Arduino and Raspberry Pi control boards, and the web server interface. A video demonstration of the system in action is accessible via the QR code. C) Petri dishes from the *P. chrysosporium* (left) and *T. reesei* (right) cultivation cycles, demonstrating the system’s effectiveness in preventing external contamination. For each cycle, the three plates represent samples from the feeding unit (left, which remained sterile), the cultivation unit (center), and the collection unit (right).

#### Arduino and Raspberry Pi Digital Controllers

An Arduino UNO microcontroller was used to control the activation of the peristaltic pumps and, consequently, the movement of the contents of each flask at defined intervals. The flow rate of the pumps was measured beforehand, and the required activation duration of each pump was estimated and programmed into the external software (Arduino IDE) used to control the system. The connection to the Raspberry Pi provided Internet access to the Arduino, allowing changes to the duration of pump activation and the size of the cultivation cycle, as well as monitoring of all stages of the process, to be carried out via a web application developed specifically for this purpose.

#### System Validation

The assembly was validated through sterility tests, confirming the absence of external contamination within the system. In the first test, conducted with LB medium for three cycles and incubated at 30 °C, no signs of contamination were detected. In the second test, performed at the end of five cycles with *P. chrysosporium*, only *P. chrysosporium* was found in the cultivation and collection flask samples, while no microorganisms were detected in the feeding container samples (Figure 1c). This result indicates not only the protection of the medium against external contamination but also the sterile integrity of the feeding unit.

### Lignin Degradation Intermediates

Under extraction with ethyl acetate, GC-MS analysis of the collected samples annotated degradation intermediate products consistent with those reported in the literature.^14,32^ Four main groups of compounds were observed in the spectral library annotation performed by GNPS (https://gnps.ucsd.edu/ProteoSAFe/status.jsp?task=cc5af8857436441f9373a6bda0a17199 ), including organic acids (e.g., lactic acid, 2-hydroxybutanoic acid, 4-hydroxybenzoic acid, palmitic acid, stearic acid), esters (e.g., bis­(2-ethylhexyl) phthalate), aromatic hydrocarbons (e.g., *m*-xylene, phenol), and alcohols (e.g., 2-ethoxyethanol, ethylene glycol).

Lignin degradation occurs in two main stages.^14^ Initially, depolymerization of lignin polymers is marked by the oxidation of lignin in the presence of air, involving the extraction of an electron from lignin, the consequent formation of free radicals from the substrate molecules, and the reduction of oxygen to water.^5^ Lignin is then degraded through catalytic polymerization and depolymerization reactions by enzymes such as laccase, caused by the instability of the formed free radicals. Furthermore, nonenzymatic reactions induced by the abundance of free radicals lead to decarboxylation, demethoxylation, and carbon–carbon chain breaks.^5^ The lignin polymer subsequently converts into oligomers that proceed to the second phase of degradation, where they are metabolized into smaller benzene-derived molecules.^14^ At this stage, the resulting free radicals replace hydrogen atoms in the side chains of aromatic compounds, forming alcohols. Consequently, a large number of aromatic alcohols are produced during lignin degradation.

From our exploratory temporal data analysis, when we performed a Principal Coordinate Analysis (PCoA) using a web interface for QIIME 2,^36^ with correlation as dissimilarity metric and Total Ion Current (TIC) normalization, the cultures clustered together based on cultivation time, while the blank samples clustered separately (Figure 2a). As PCoA represents a lower dimension projection of the weighted contributions of all detected metabolites, this indicates that the metabolomes obtained for each culture could be distinguished from the initial set of lignin components. The 20-h time point for *P. chrysosporium* deviated from the others, suggesting a possible processing outlier. We also observed that the coculture exhibited a pattern closer to *T. reesei*, implying a stronger metabolic influence from this species on the overall composition (Figure S8 and Table S5). The presence of several small aromatic compounds was noted, such as dimethyl phthalate, 4-hydroxybenzoic acid, benzoic acid and 3-tert-butylphenol—all four described in lignin degradation processes (Figure 2b).

**Figure 2:**
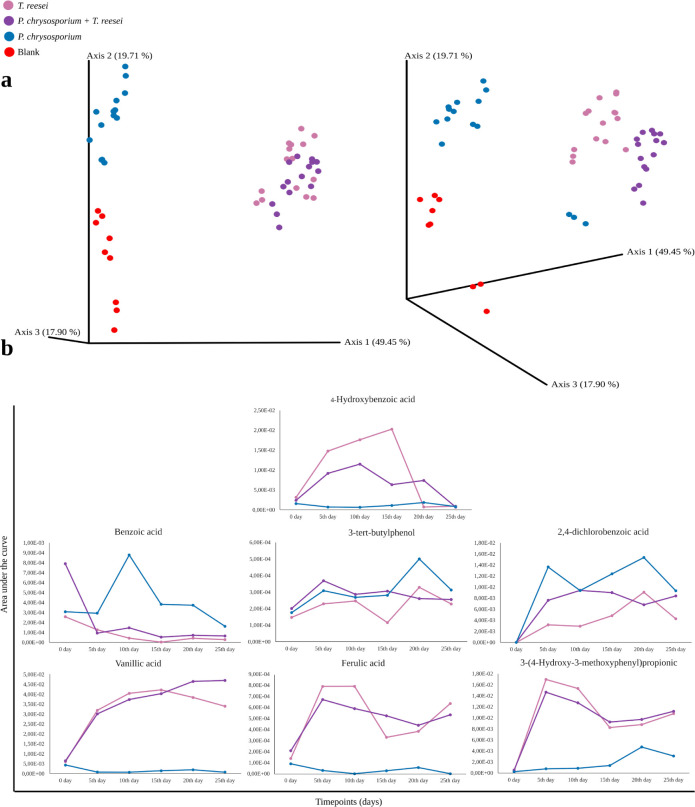
Monitoring of compound levels over a 25-day period. A) Principal Coordinate Analysis (PCoA) performed with a web interface for QIIME 2. B) Graphs showing the growth and decline patterns of the area under the curve for key compounds involved in lignin degradation. The *y*-axis represents raw area under the curve ion intensity.

Benzoic acid and its derivative 4-hydroxybenzoic acid are central intermediates in several lignin degradation pathways, such as the gentisic acid pathway, coumaric acid pathway, and cinnamic acid pathway.^33^ In the temporal analysis, benzoic acid peaks were observed across all three culture conditions (Figure 2b). However, as the current version of the bioreactor enables only a single culture by time series, the quantitative fluctuation patterns could not be confirmed by our validation experiment (Table S4 and Figure S3), therefore, all the following discussion will be centered around the consistency of the detection of metabolites knowingly associated with lignin degradation, as demonstrated by the low standard deviation of the metabolite signal detected by GC-MS over time (Figure S7). The 4-hydroxybenzoic acid was also consistently detected in the *T. reesei* monoculture and the coculture. This intermediate is produced by the activity of benzoate 4-monooxygenase, an enzyme present in the *T. reesei* genome^34^ that can convert benzoic acid into 4-hydroxybenzoic acid.

Tert-butylphenol derivatives have been the focus of recent studies that demonstrate their involvement in microbial lignin degradation pathways, such as the resorcinol pathway.^35^ Considering the consistent detection of this molecule over time, it can be suggested that *P. chrysosporium*, *T. reesei*, or their combination may possess a similar pathway, where the tert-butyl structure is retained and metabolized via the meta-cleavage pathway (Figure 2b).

#### Chlorinated Compounds

Due to the lignin being sourced from pulp and paper industry waste, a significant number of chlorinated compounds were detected in the analyses. Among these, 2,4-dichlorobenzoic acid was particularly noteworthy (Figure 2b). This could be attributed to the chlorination of the lignin polymeric structure, a process commonly employed in the paper and cellulose industries.^6^ To investigate whether chlorine was present before the cultivation, we inspected our culture media control (labeled *BLANK*) samples and observed that those compounds were present before cultivation (Table S6). This benzoic acid derivative likely resulted from reactions similar to those that produced compounds such as 4-hydroxybenzoic acid.

Considering only the compounds reported in the literature as lignin degradation products,^37–39^ the coculture enabled the detection of 11 compounds, including guaiacol, adipic acid, vanillic acid, and malonic acid. In comparison, *P. chrysosporium* and *T. reesei* each afforded the detection of 4 such compounds. Among these, 2-methoxy-5-methylphenol was common to both the *P. chrysosporium* monoculture and the coculture, while adipic acid was found in both the *T. reesei* monoculture and the coculture.

The analysis reveals a diverse metabolic response within the coculture, as indicated by the increased number of compounds detected. This suggests a potential synergistic interaction between the two species, particularly in relation to lignin degradation. However, this hypothesis would need further investigation to be confirmed, for example, with a quantitative PCR (qPCR) assay to estimate the biomass of each species.^43^ We also performed a cocultivation in solid medium (Figure S4) to inspect if the inhibition of one species by the other could be detected, and no sign of a growth exclusion zone was observed. Such interactions likely lead to the development of unique and distinct metabolic pathways compared to those observed in monoculture.

In terms of exclusivity, three annotated compounds (*N*-acetyl-leucine, phosphine, 1,3-propanediylbis­[dicyclohexyl]-phosphine, and 2-(3-methylbutoxy)-4-(trifluoromethyl))-1,3-dithiaindane were detected in both the *T. reesei* monoculture and the coculture, but were absent in the *P. chrysosporium* monoculture. Notably, no compounds were found to be exclusive to either *P. chrysosporium*, *T. reesei*, or the coculture alone (Figure S6). This absence of exclusive compounds across the conditions suggests a shared metabolic environment, where certain metabolites are either consistently produced or inhibited depending on the presence of specific interactions between the species.

When focusing on compounds typically associated with the final stages of lignin degradation,^37–41^ the main compounds detected in our experiment are outlined in Figure 3.

**Figure 3:**
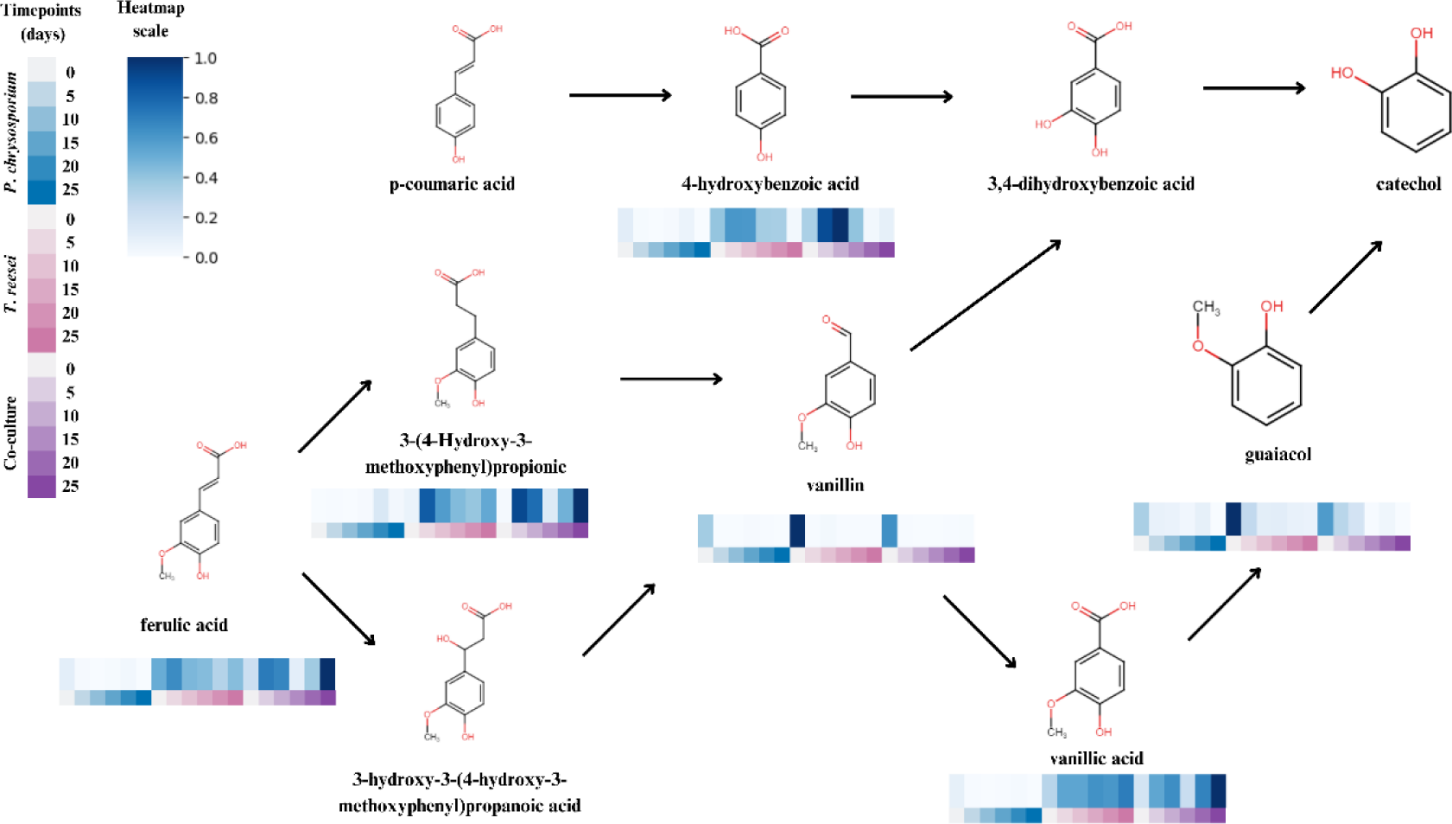
Proposed final stages of lignin degradation, highlighting the hypothetical reactions that annotated compounds may undergo within the system. The accompanying heatmap illustrates the variation in the areas under the curve for these compounds across different time points, indicating at which time points the compounds were detected, without the possibility of direct quantitative interpretation.

To further illustrate the behavior of the compounds associated with lignin metabolism, we investigated spectrally similar compounds in our study using the molecular network obtained from GNPS. As an initial validation of our spectral annotation, the spectral annotations of two known lignin intermediates, ferulic acid and vanillic acid, were confirmed by acquiring spectra under the same spectrometric conditions and comparing them to our experimental spectra (Figure S5), yielding cosine scores greater than 0.95. The detected vanillin may have been subsequently oxidized to vanillic acid, as indicated by the clustering in the network (Figure 4a). Isovanillic acid, which was also annotated (Figure 4a), can be considered a methylated isomer of vanillin, formed as an alternative product through regioselective methylation reactions.

**Figure 4:**
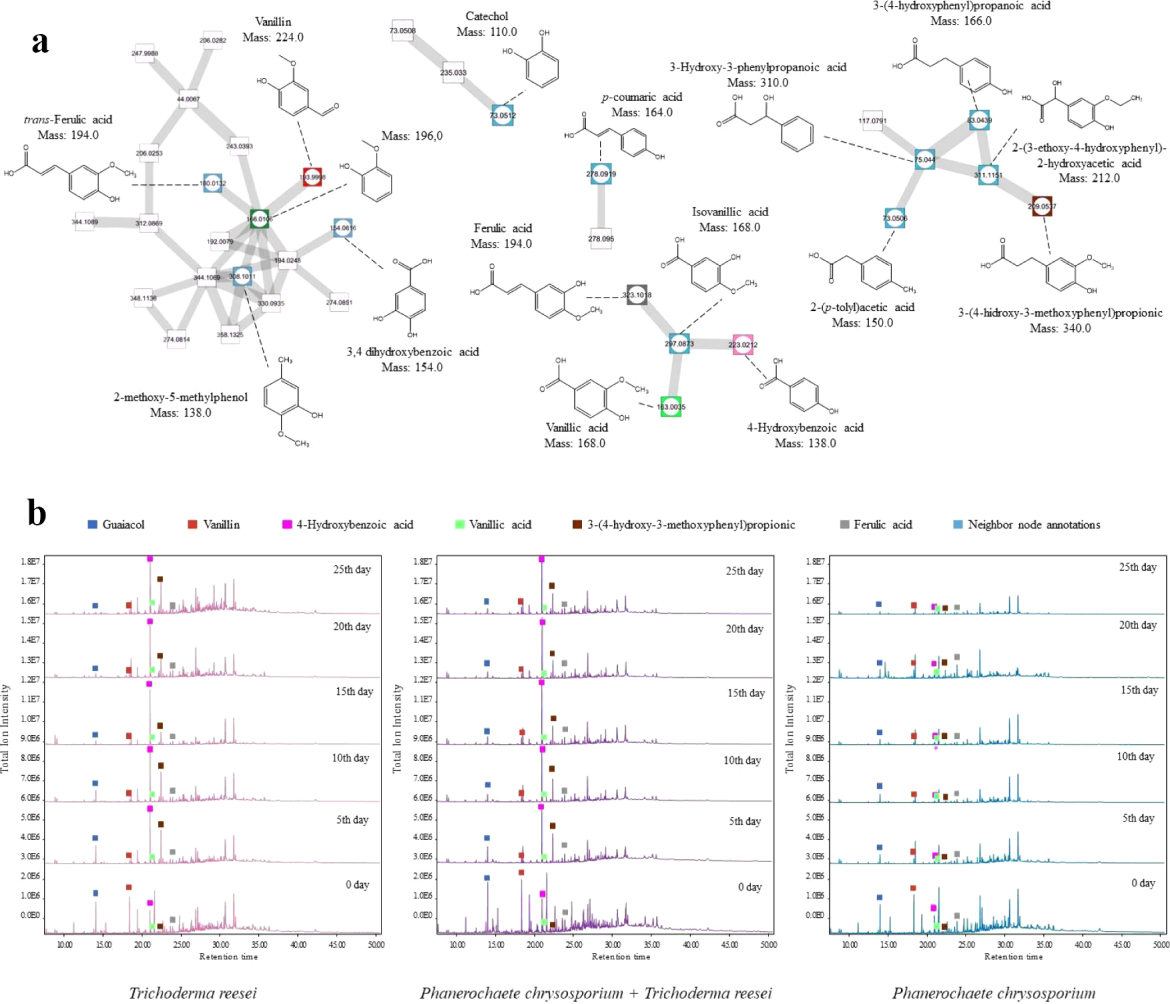
GC-MS/MS analysis of lignin degradation intermediates produced by *P. chrysosporium*, *T. reesei*, and coculture. a) Molecular network (Cytoscape) of metabolite interactions based on structural similarity (GNPS). Blue nodes indicate annotated neighbor compounds; differently colored nodes correspond to compounds shown in Figure 3. Complete data sets are available in Table S1 and Figure S1 (Supporting Information). b) Comparative chromatograms showing temporal progression of metabolite production across culture conditions. Peaks marked with squares (■) represent identified lignin degradation intermediates, with corresponding retention times (RT). The *y*-axis represents raw area under the curve ion intensity

The detection of 4-hydroxybenzoic acid suggests oxidative demethoxylation of precursors such as vanillin or vanillic acid, while also potentially representing an intermediate step in aromatic ring catabolism. The identification of catechol in the molecular network (Figure 4a) further supports this degradative pathway, which likely forms through the oxidative decarboxylation of aromatic intermediates.

Ferulic acid, in turn, may have been converted to vanillin. p-Coumaric acid may serve as a direct precursor to ferulic acid via phenolic ring methoxylation. The detection of compound 3-(4-hydroxy-3-methoxyphenyl)­propionic acid may suggest the reductive modification of ferulic acid’s side chain. Its absence in coculture implies either cross-consumption or metabolic repression by *P. chrysosporium*. Additionally, compounds such as 2-(3-ethoxy-4-hydroxyphenyl)-2-hydroxyacetic acid were observed, indicating possible ethoxylation and acyl hydrolysis reactions, common under coculture conditions, which promote structural diversification of the aromatic matrix.

The detected guaiacol may suggest the depolymerization of lignin’s guaiacyl units (Figure 4b). Its formation is consistent with oxidative demethylation of lignin subunits. Furthermore, the presence of derivatives such as 2-methoxy-5-methylphenol suggests that aromatic ring methylation and rearrangement occur as subsequent steps following guaiacol release.

While the observed patterns may result from metabolic interactions between the two fungi, methodological limitations warrant consideration, as our sampling frequency may have missed transient metabolic intermediates or rapid dynamic events in this interaction. Future work could address these through higher-resolution sampling (e.g., daily or hourly).

### Lignin Degradation Enzymes in *Phanerochaete chrysosporium* and*Trichoderma reesei*

Since preliminary annotation of the metabolic dynamics of lignin degradation pointed to known enzymatic transformations of this biopolymer, we investigated the presence of genes encoding the corresponding enzymes in the genomes of *P. chrysosporium* and *T. reesei*. We first selected enzymatic reactions capable of producing the transformations observed in Figure 2, such as *p*-hydroxybenzoate-*m*-hydroxylase activity. With the selected enzymatic reactions, we obtained representative fungi sequences from Swiss-Prot as reference in our annotation workflow. Standalone tBLASTn relaxed search, translating all coding sequences predicted by Augustus in all six frames and comparing with reference proteins, resulted in 675 unique candidate regions for lignin-degrading enzyme genes. We filtered these matches through BLASTp analysis, comparing the Swiss-Prot enzymes against the hypothetical proteins encoded by the candidate genes. Four different genes predicted by Augustus met the DIAMOND *e*-value cutoff, and three of them showed significant alignment based on bit score analysis (Table S2).

BLASTp analysis using the UniProt Web server revealed that g7178 shares 88% identity with an in silico-annotated FAD-binding domain-containing protein (UniProt ID: K5XCV5) in *Phanerochaete carnosa*. Moreover, standalone BLASTp g7179 analysis showed also 33.6% and 35.1% similarity to two *p*-hydroxybenzoate-*m*-hydroxylases: hydroxylase A from *Aspergillus niger* (Swiss-Prot ID: A2QGH7) and *p*-hydroxybenzoate-*m*-hydroxylase from *Emericella nidulans* (formerly *Aspergillus nidulans*; Swiss-Prot ID: C8VBV0), respectively. These FAD-dependent monooxygenases are known to catalyze the conversion of 4-hydroxybenzoate (Figure 2) to 3,4-dihydroxybenzoate.

Similarly, g1293 presents 89.9% similarity to an in silico-annotated aldehyde dehydrogenase (UniProt ID: A0A9P3G6L2) from *Phanerochaete sordida*, as well as high coverage but low identity with YfmT, a benzaldehyde dehydrogenase from *Bacillus subtilis* (Swiss-Prot ID: O06478) that converts vanillin to vanillic acid, and two vanillin dehydrogenases from *Corynebacterium glutamicum* (Swiss-Prot ID: Q8NMB0) and *Pseudomonas* sp. (Swiss-Prot ID: O05619).

To obtain additional evidence for the enzymatic functions annotated, we further enquired if these genes would be expressed in the presence of lignin as a carbon source in an independent experimental setting. To assess that, public RNA-Seq data from an experiment that measured the gene expression of *P. chrysosporium* after 40h and 96h of cultivation^42^ were reanalyzed and integrated into our results. Differential gene expression analysis showed the expression of g1293 and g7178 differed significantly from 40h to 96h, providing additional evidence that these candidates encode enzymes involved in lignin degradation.

We applied the same workflow to *T. reesei* QM6a (assembly GCF_000167675.1), except for the gene prediction step, since a protein FASTA file was already available. Notably, 81% of the 9118 protein sequences predicted by the NCBI pipeline were tagged as uncharacterized. Our standalone tBLASTn analysis showed 859 matches between Swiss-Prot proteins and predicted genes in the *T. reesei* QM6a genome. Subsequent standalone BLASTp comparisons selected four predicted proteins showing significant alignment against Swiss-Prot proteins (Table S3).

The protein XP_006968037.1 shows 98% similarity to an in silico-predicted FAD-binding domain-containing protein (UniProt ID: A0A2T4BXX9) from *Trichoderma longibrachiatum* ATCC 18648. Also, similar to g7178, XP_006968037.1 exhibits the highest percentage identity and bit score values when aligned with two *p*-hydroxybenzoate-*m*-hydroxylases. Another similarity between *T. reesei* and *P. chrysosporium* is observed through XP_006964001.1, which showed significant matches to three vanillin dehydrogenases.

It is worth noting that, despite these strong in silico correlations, further investigation, including biochemical or expression-based validation, is required to confirm the proposed enzymatic functions.

### Protein Sequence and Structure Analysis

Sequence alignments revealing matches with low identity and high coverage suggested that both fungi possess unannotated *p*-hydroxybenzoate-*m*-hydroxylases and vanillin dehydrogenases. Therefore, we investigated whether Swiss-Prot enzymes and our candidate proteins shared functional domains and/or structural similarities. Structural alignments of 3D models supported functional predictions; for example, vanillin dehydrogenase candidate g1293 (*P. chrysosporium*) presented RMSD values of 1 Å, 0.877 Å and 0.959 Å when aligned with reference structures O05619, O06478, and Q8NMB0, respectively. Similarly, XP_006964001.1 (*T. reesei*) showed RMSD values of 0.858 Å and 1.031 Å against Q8NMB0 and O05619.

Despite sequence-level divergence between *p*-hydroxybenzoate-*m*-hydroxylase candidates XP_006968037.1 (*T. reesei*) and g7178 (*P. chrysosporium*) relative to reference C8VBV0, their low values of RMSD indicate strong structural conservation (Figure 5A,B). For example, the FAD-binding domain (InterPro ID PF01565) remained structurally intact even in the most divergent sequence, g7178 (Figure 5C). Additional support came from g7178 and XP_006968037.1, which demonstrated structural similarity (RMSD = 0.834 Å and 0.526 Å, respectively) to reference A2QGH7.

**Figure 5:**
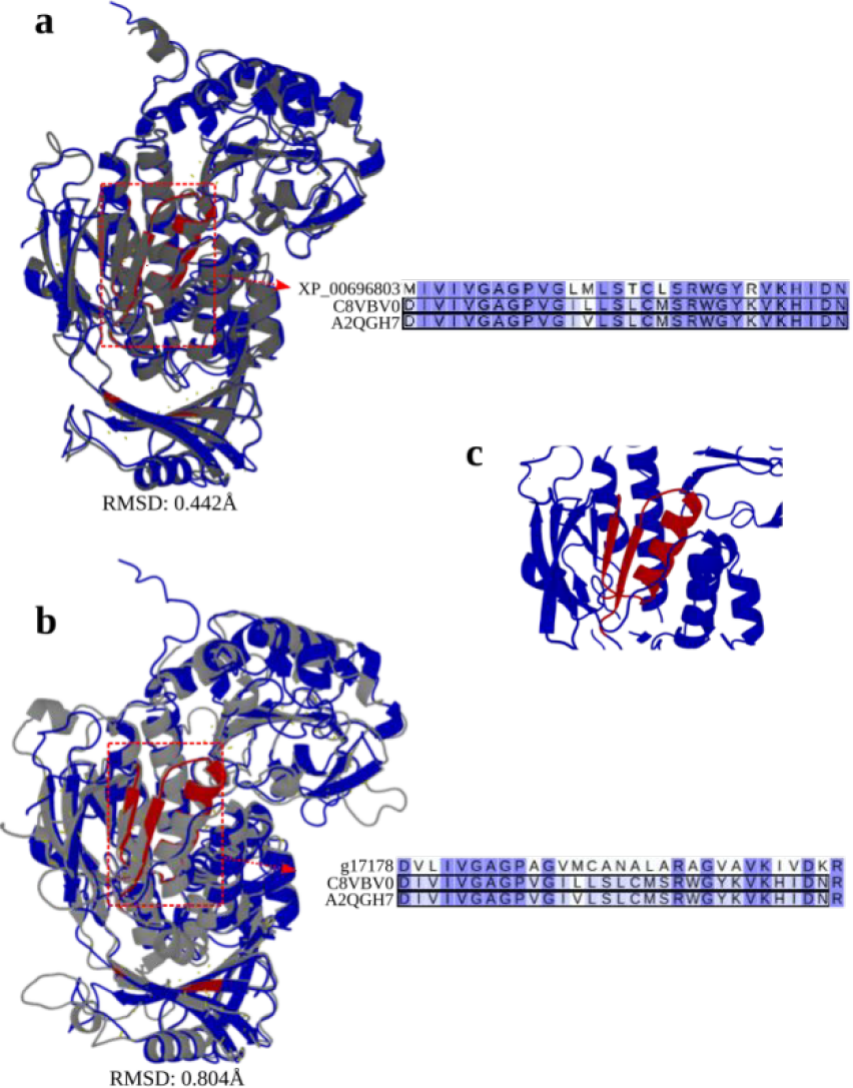
Structural and FAD-binding domain comparison among *p*-hydroxybenzoate-*m*-hydroxylases. A) Structural alignment between XP_006968037.1 (dark gray, *T. reesei*) and reference protein C8VBV0 (blue). B) Structural alignment between g7178 (light gray, *P. chrysosporium*) and reference protein C8VBV0 (blue). The letter C and the sequence alignment represent FAD-binding domains.

As noted, these enzymatic annotations are based solely on in silico evidence; biochemical or expression-based validation remains necessary for confirmation.

## Conclusions

Overall, the results of this investigation suggest a potential synergistic interaction between *P. chrysosporium* and *T. reesei* in lignin degradation. However, further studies are needed to deepen our understanding of this interaction and to explore ways to optimize it. Genomic analyses generated valuable insights into the molecular mechanisms underlying lignin metabolism, providing potential targets for enhancing ligninolytic activity through genetic engineering or adaptive laboratory evolution (ALE), which can be streamlined by using an automated bioreactor. Additionally, investigations that explore variations in environmental conditions or cultivation periods may offer strategies to increase enzyme production and enhance the lignin degradation capabilities of these species. Our open-source bioreactor project can be expanded to test a wide range of culture conditions, offering new insights into the metabolic dynamics. Such research could contribute significantly to the development of more efficient biotechnological applications for lignin valorization.

## Supplementary Material



## Notes

Additional tables; figures; GNPS job link for complementary experiments described in the manuscript (PDF)
