# Supplementary material for: Semiautomated Monitoring of Longitudinal Microbial Metabolic Dynamics: A Study Case for Lignin Degradation
Source: ACS Omega. 2026 Jan 26;11(5):7022–32. doi: 10.1021/acsomega.5c05607 (PMC12903019; doi:10.1021/acsomega.5c05607)
Supplement: Supplementary file 1 [file ao5c05607_si_001.pdf]

# Supporting Information

# Semi-automated monitoring of longitudinal microbial metabolic dynamics: A study case for lignin degradation

**Authorship:** João Vítor Guimarães Ferreira, Gabriel Santos Arini, Tiago Cabral Borelli, Isabela Victorino da Silva Amatto, Winner Duque Rodrigues, Igor Sepulveda Rodrigues, Nathália Gonsales da Rosa-Garzon, Izabel Cristina Casanova Turatti, Henrique Marcel Yudi de Oliveira Tsuji, Iasmin Cartaxo Taveira, Livia Soares Zaramela, Norberto Peporine Lopes, Hamilton Cabral, Ricardo Roberto da Silva*

**Contact information:** Faculty of Pharmaceutical Sciences of Ribeirão Preto, University of São Paulo, Ribeirão Preto, São Paulo, Brazil
*Email: ridasilva@usp.br

Table S1. GNPS data on lignin degradation intermediates.

| Compound name | Scan | MQ Score | Spectrum ID | RT Query |
|---|---|---|---|---|
| Ferulic acid | 418 | 0.74 | CCMSLIB00005458141 | 24.1363 |
| 3-(4-Hydroxy-3-methoxyphenyl)propionic | 335 | 0.92 | CCMSLIB00001021726 | 22.1877 |
| Vanillic acid | 284 | 0.83 | CCMSLIB00005459280 | 20.8120 |
| 4-Hydroxybenzoic acid | 281 | 0.76 | CCMSLIB00005457969 | 20.8051 |
| Vanillin | 207 | 0.94 | CCMSLIB00000886153 | 18.1978 |
| Guaiacol | 103 | 0.90 | CCMSLIB00005460664 | 13.9178 |

Table S2. Annotation for enzymes associated with lignin metabolism for *Phanerochaete chrysosporium.*

| Swiss-Prot | Augustus | Augustussscore | Identity (%) | E-value | Bit Score | *Start | End | Length | EC number |
|---|---|---|---|---|---|---|---|---|---|
| O06478 | g1293 | 0.95 | 33.7 | 2.27e-76 | 244.0 | 25 | 510 | 516 | 1.2.1.67 |
| Q8NMB0 | g1293 | 0.95 | 34.9 | 4.64e-75 | 241.0 | 25 | 510 | 516 | 1.2.1.67 |
| O05619 | g1293 | 0.95 | 33.9 | 3.05e-75 | 241.0 | 24 | 513 | 516 | 1.2.1.67 |

| | | | | | | | | | |
|---|---|---|---|---|---|---|---|---|---|
| K9UV87 | g6547 | 0.39 | 34.5 | 4.54e-64 | 211.0 | 44 | 500 | 502 | 1.2.1.67 |
| P20586 | g9016 | 0.71 | 24.6 | 2.1e-05 | 41.6 | 8 | 356 | 441 | 1.14.13.2 |
| P00438 | g9016 | 0.71 | 24.6 | 2.78e-05 | 41.2 | 8 | 356 | 441 | 1.14.13.2 |
| A2QGH7 | g7178 | 0.79 | 33.6 | 1.69e-109 | 338.0 | 4 | 605 | 609 | 1.14.13.33 |
| C8VBV0 | g7178 | 0.79 | 35.1 | 1.98e-115 | 353.0 | 12 | 605 | 609 | 1.14.13.33 |

*The columns Start, and End and Length refer to the hypothetical proteins annotated by Augustus

Table S3. Annotation for enzymes associated with lignin metabolism for *Trichoderma reesei.*

| **Swiss-Prot** | **Protein Id** | **Identity (%)** | **E-value** | **Bit Score** | **Start*** | **End** | **Length** | **EC number** |
|---|---|---|---|---|---|---|---|---|
| Q8NMB0 | XP_006964001.1 | 30.3 | 9.18e-45 | 158.0 | 4 | 423 | 444 | 1.2.1.67 |
| O05619 | XP_006964001.1 | 29.5 | 3.33e-46 | 162.0 | 19 | 423 | 444 | 1.2.1.67 |
| K9UV87 | XP_006964464.1 | 30.8 | 1.02e-52 | 181.0 | 42 | 473 | 494 | 1.2.1.67 |
| P20586 | XP_006969393.1 | 26.6 | 2.41e-06 | 44.3 | 1 | 327 | 504 | 1.14.13.2 |
| P00438 | XP_006969393.1 | 26.6 | 3.19e-06 | 43.9 | 1 | 327 | 504 | 1.14.13.2 |
| A2QGH7 | XP_006968037.1 | 62.6 | 3.64e-291 | 803.0 | 2 | 627 | 628 | 1.14.13.33 |
| C8VBV0 | XP_006968037.1 | 63.2 | 2.03e-295 | 813.0 | 2 | 626 | 628 | 1.14.13.33 |
| C4TP09 | XP_006969393.1 | 56.3 | 8.49e-04 | 36.2 | 271 | 302 | 504 | 1.14.13.33 |

*The columns Start, and End and Length refer to the hypothetical proteins annotated by Augustus

Table S4. GNPS annotation (https://gnps.ucsd.edu/ProteoSAFe/status.jsp?task=2e18e073ede741fc91d9a26ab65c7fb4) data on lignin degradation intermediates for the validation experiment, with its respective correlation as well as p-values for all experimental conditions.

| **SpectrumID** | **Compound_Name** | **#Scan#** | **MQScore** | **RT_Query** | **Corr *P. chrysosporium*** | **p-value *P. chrysosporium*** | **Corr *T. reesei*** | **p-values *T. reesei*** | **Corr Co-culture** | **p-values cultur** |
|---|---|---|---|---|---|---|---|---|---|---|
| CCMSLIB00000886152 | Isovanillin, TMS derivative | 76 | 0,954328 | 1106,81 | -0,4796810699 | 0,4135560794 | -0,4231812213 | 0,4777331714 | -0,5867306322 | 0,298356 |
| CCMSLIB00000886152 | Isovanillin, TMS derivative | 64 | 0,674853 | 994,476 | -0,3141133193 | 0,6067362925 | -0,6189909239 | 0,2655880614 | -0,02107839938 | 0,973164 |
| CCMSLIB00000886153 | Vanillin, TMS derivative | 76 | 0,953981 | 1106,81 | -0,4796810699 | 0,4135560794 | -0,4231812213 | 0,4777331714 | -0,5867306322 | 0,298356 |
| CCMSLIB00000886153 | Vanillin, TMS derivative | 64 | 0,675479 | 994,476 | -0,3141133193 | 0,6067362925 | -0,6189909239 | 0,2655880614 | -0,02107839938 | 0,973164 |
| CCMSLIB00000886220 | Vanillin, TBDMS derivative | 76 | 0,891377 | 1106,81 | -0,3141133193 | 0,6067362925 | -0,6189909239 | 0,2655880614 | -0,02107839938 | 0,973164 |
| CCMSLIB00000886221 | Isovanillin, TBDMS derivative | 76 | 0,894079 | 1106,81 | -0,3141133193 | 0,6067362925 | -0,6189909239 | 0,2655880614 | -0,02107839938 | 0,973164 |
| CCMSLIB00000942684 | Vanillic Acid, 2TMS derivative | 97 | 0,840361 | 1259,23 | -0,438392989 | 0,4602545022 | 0,05053875566 | 0,9356794608 | -0,8126172313 | 0,094586 |
| CCMSLIB00000942684 | Vanillic Acid, 2TMS derivative | 96 | 0,706049 | 1259,43 | -0,1528733857 | 0,8061163891 | 0,00157465224 | 0,9979950913 | -0,7656638658 | 0,131281 |
| CCMSLIB00001001095 | Ferulic acid, 2TMS derivative | 135 | 0,660182 | 1459,64 | -0,3301868529 | 0,5873621356 | -0,2059713861 | 0,7396153668 | -0,8723959103 | 0,053658 |
| CCMSLIB00001001178 | Isoferulic acid, 2TMS derivative | 135 | 0,706567 | 1459,64 | -0,3301868529 | 0,5873621356 | -0,2059713861 | 0,7396153668 | -0,8723959103 | 0,053658 |
| CCMSLIB00001005131 | Isovanillic acid, 2TMS derivative | 97 | 0,886579 | 1259,23 | -0,1528733857 | 0,8061163891 | 0,00157465224 | 0,9979950913 | -0,7656638658 | 0,131281 |

| | | | | | | | | | | |
|---|---|---|---|---|---|---|---|---|---|---|
| CCMSLIB00001008607 | 4-Hydroxybenzoic acid, 2TMS derivative | 96 | 0,698515 | 1259,43 | -0,438392989 | 0,4602545022 | 0,05053875566 | 0,9356794608 | -0,8126172313 | 0,094586 |
| CCMSLIB00001021726 | 3-(4-Hydroxy-3-methoxyphenyl)propionic acid, 2TMS derivative | 107 | 0,88069 | 1342,41 | -0,2616698479 | 0,6706737081 | -0,02794196045 | 0,964427821 | -0,7724089509 | 0,125792 |
| CCMSLIB00001022024 | o-Vanillin, TBDMS derivative | 76 | 0,822252 | 1106,81 | -0,3141133193 | 0,6067362925 | -0,6189909239 | 0,2655880614 | -0,02107839938 | 0,973164 |
| CCMSLIB00001022025 | o-Vanillin, TMS derivative | 76 | 0,876806 | 1106,81 | -0,3141133193 | 0,6067362925 | -0,6189909239 | 0,2655880614 | -0,02107839938 | 0,973164 |
| CCMSLIB00001038508 | Guaiacol, TBDMS derivative | 47 | 0,880401 | 847,82 | -0,1825780137 | 0,7688325185 | -0,2244837845 | 0,7165974158 | -0,4993901119 | 0,391674 |
| CCMSLIB00001038509 | Guaiacol, TMS derivative | 47 | 0,953082 | 847,82 | -0,1825780137 | 0,7688325185 | -0,2244837845 | 0,7165974158 | -0,4993901119 | 0,391674 |
| CCMSLIB00005448669 | HOMOVANILLIC ACID-DI-TRIMETHYLSILYL ETHER | 643 | 0,698502 | 2327,06 | 0,6128014177 | 0,2717966855 | -0,3796065159 | 0,5285428478 | -0,06577348998 | 0,916315 |
| CCMSLIB00005457909 | Homovanillic acid | 100 | 0,655193 | 1293,76 | -0,7728709364 | 0,1254190568 | -0,4499189398 | 0,4471062909 | -0,6386142467 | 0,246161 |
| CCMSLIB00005457969 | 4-Hydroxybenzoic acid | 97 | 0,654112 | 1259,23 | -0,1528733857 | 0,8061163891 | 0,00157465224 | 0,9979950913 | -0,7656638658 | 0,131281 |
| CCMSLIB00005459280 | Vanillic acid | 97 | 0,83291 | 1259,23 | -0,1528733857 | 0,8061163891 | 0,00157465224 | 0,9979950913 | -0,7656638658 | 0,131281 |
| CCMSLIB00005460664 | guaiacol | 47 | 0,893403 | 847,82 | -0,1825780137 | 0,7688325185 | -0,2244837845 | 0,7165974158 | -0,4993901119 | 0,391674 |
| CCMSLIB00005460845 | homovanillic acid | 100 | 0,654001 | 1293,76 | -0,7728709364 | 0,1254190568 | -0,4499189398 | 0,4471062909 | -0,6386142467 | 0,246161 |

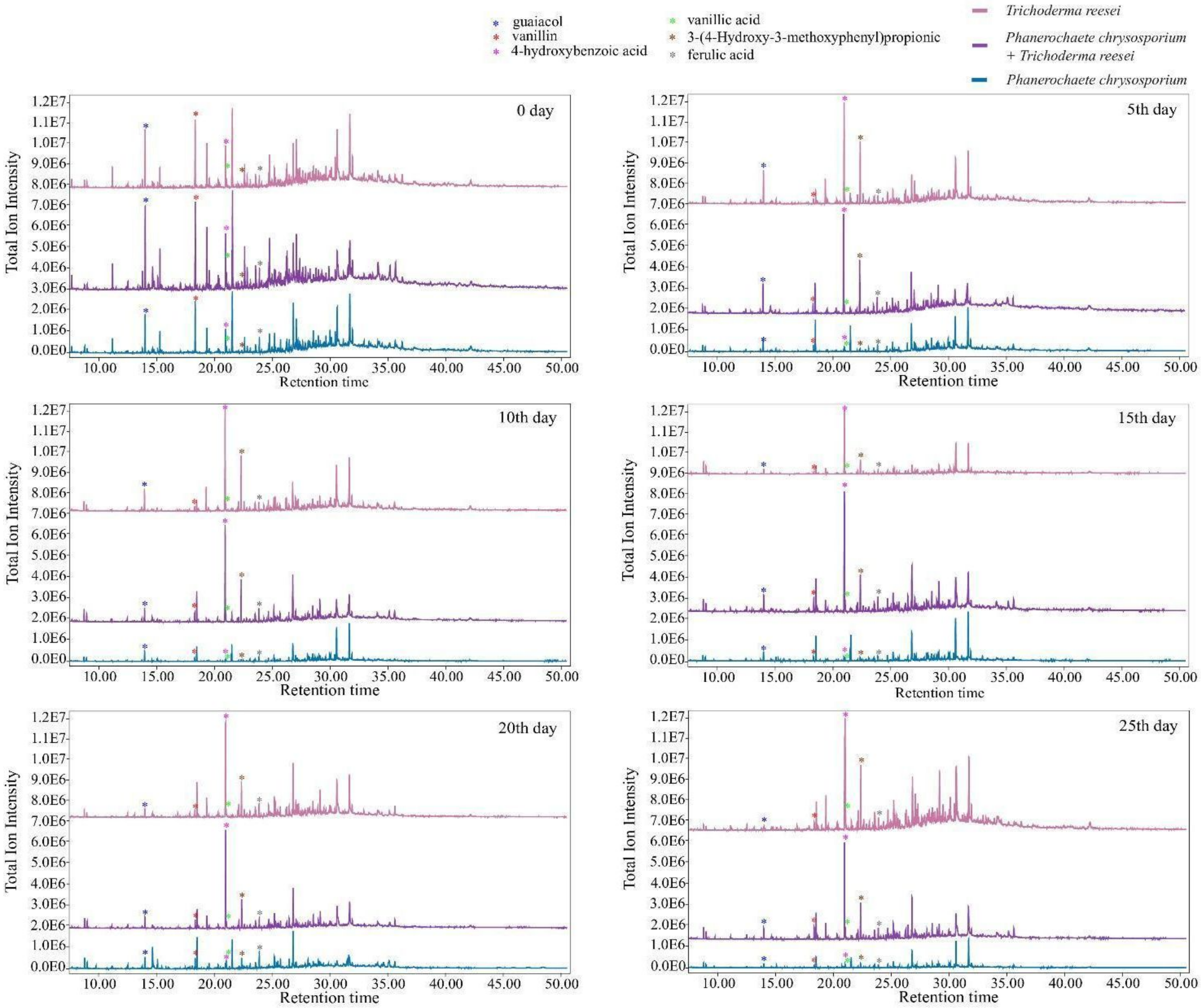


Figure S1. Chromatographic profiles segmented by incubation time for cultures of *P. chrysosporium*, *T. reesei*, and the co-culture, highlighting lignin degradation intermediates based on their respective retention times.

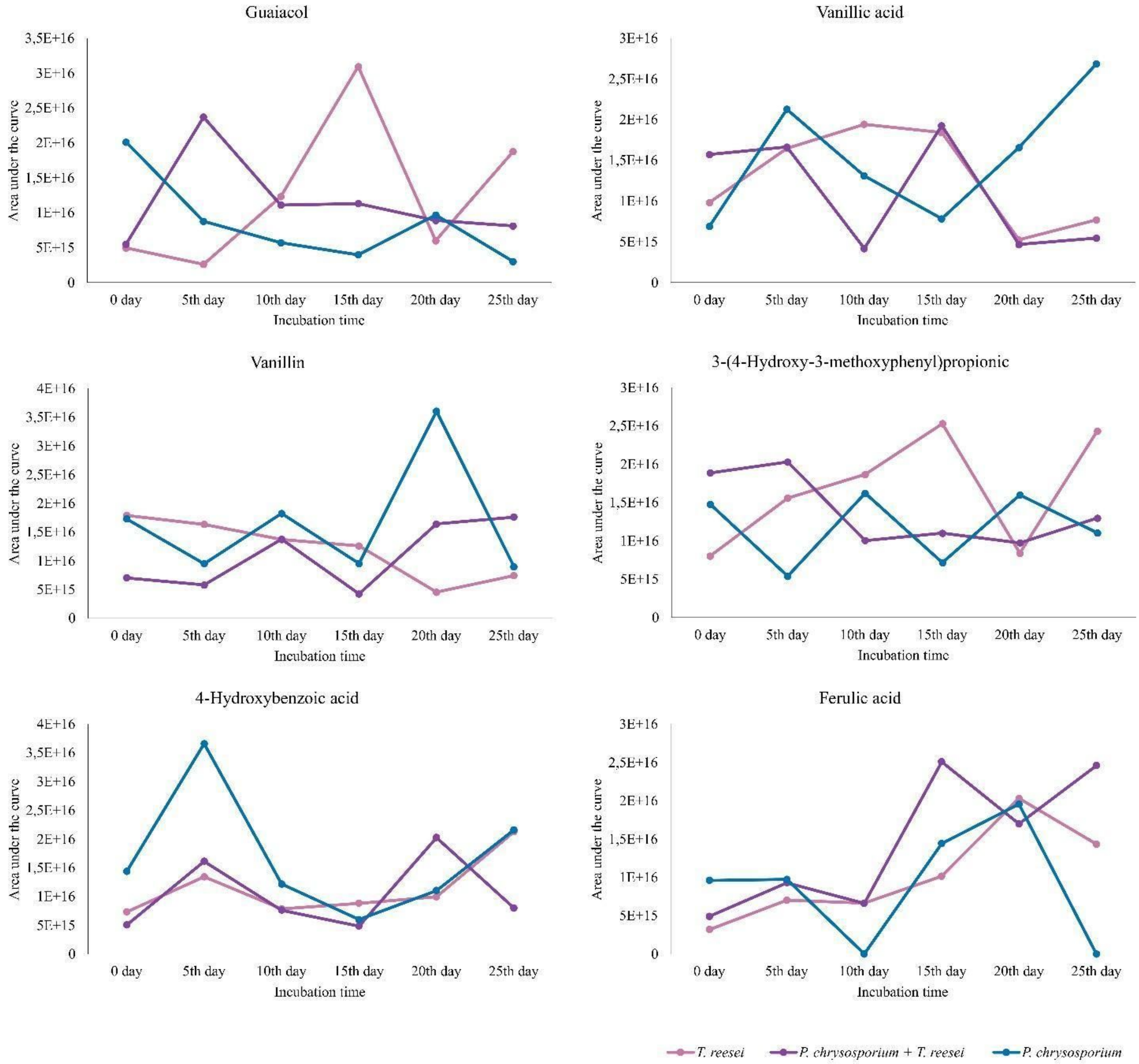


Figure S2. Time-course analysis of lignin degradation intermediates in cultures of *P. chrysosporium*, *T. reesei*, and the co-culture, represented by the area under the curve (AUC) for each detected compound.

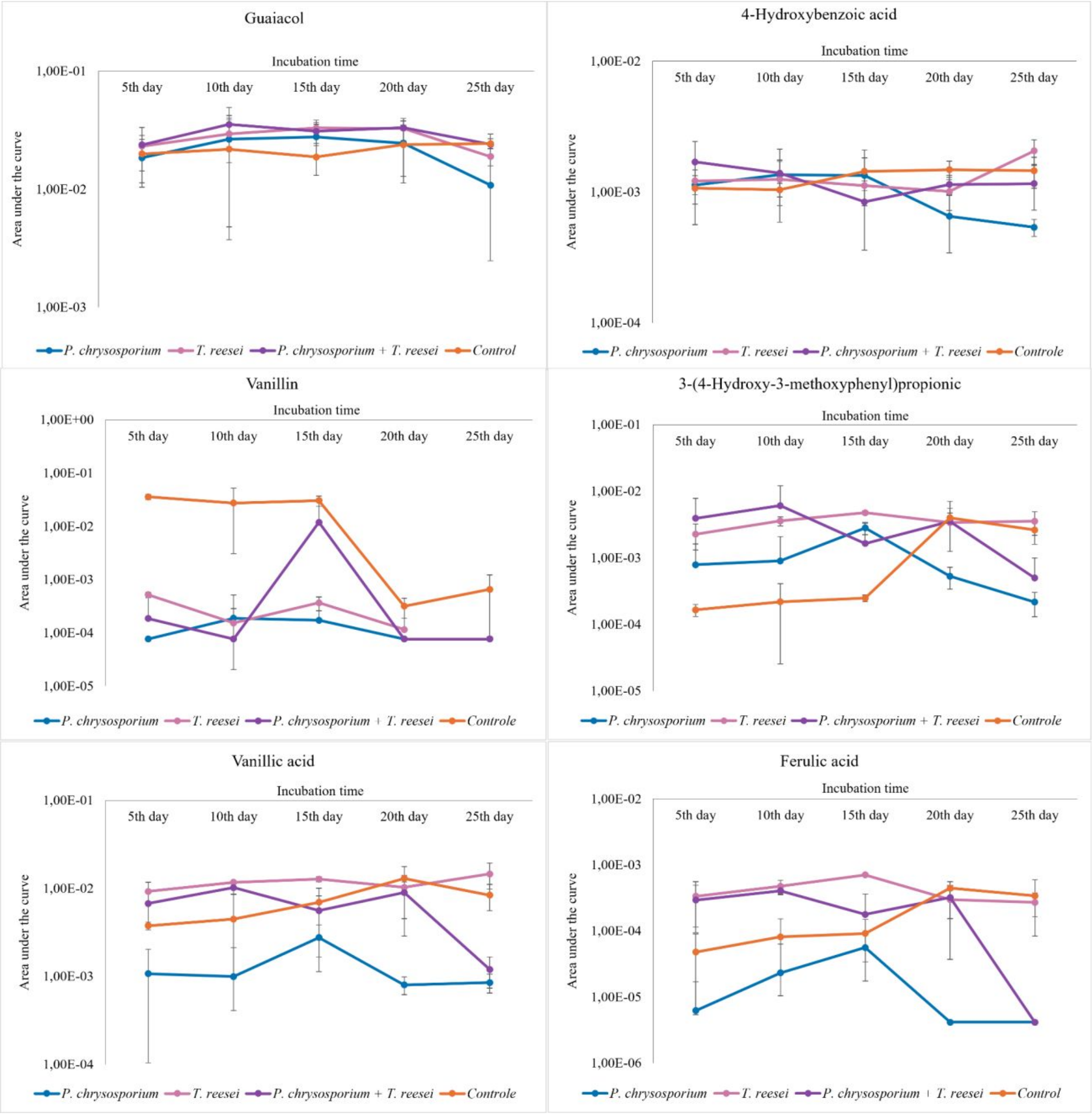


Figure S3. Time-course analysis with 3 replicates of lignin degradation intermediates in cultures of *P. chrysosporium*, *T. reesei*, the co-culture, and control, represented by the area under the curve (AUC) for each detected compound.

* On the 13th day of the experiment, the shaker remained off due to an electricity outage from 18:00 (6 pm) of 08/11/25 to 10:00 (10 am) of 08/12/25. The experiment was continued, and fungal growth was visually observed in the following days.

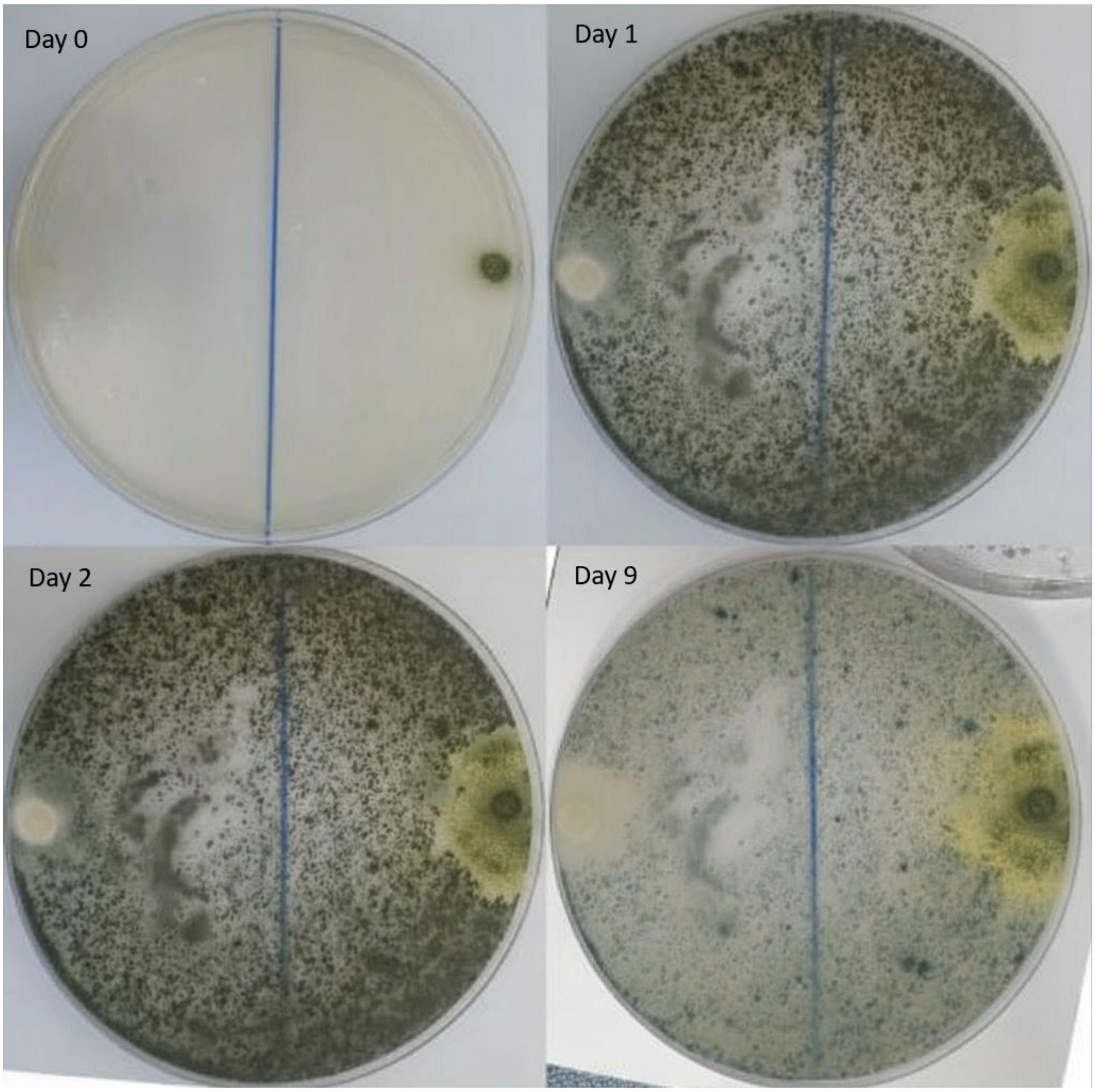


Figure S4. Co-cultivation of *P. chrysosporium* (right) and *T. reesei* (left) showing no sign of growth exclusion zone.

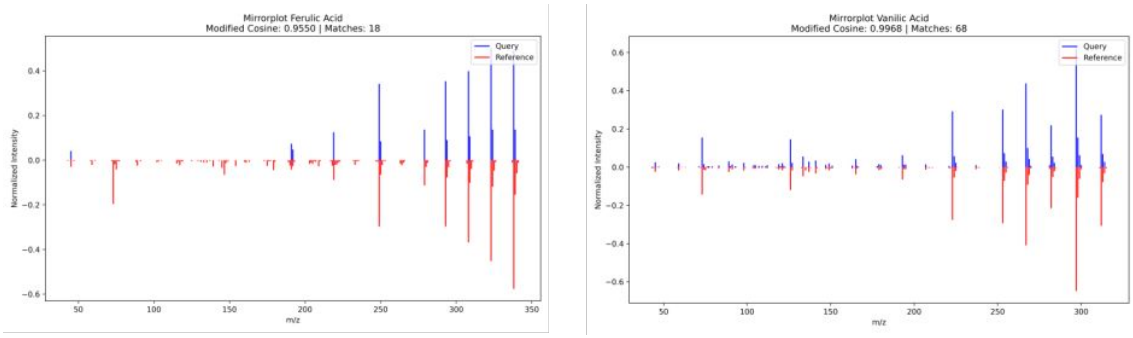


Figure S5. Spectral comparison of commercial standards (Reference) to ions detected in the experiment (Query) for ferulic acid and vanillic acid.

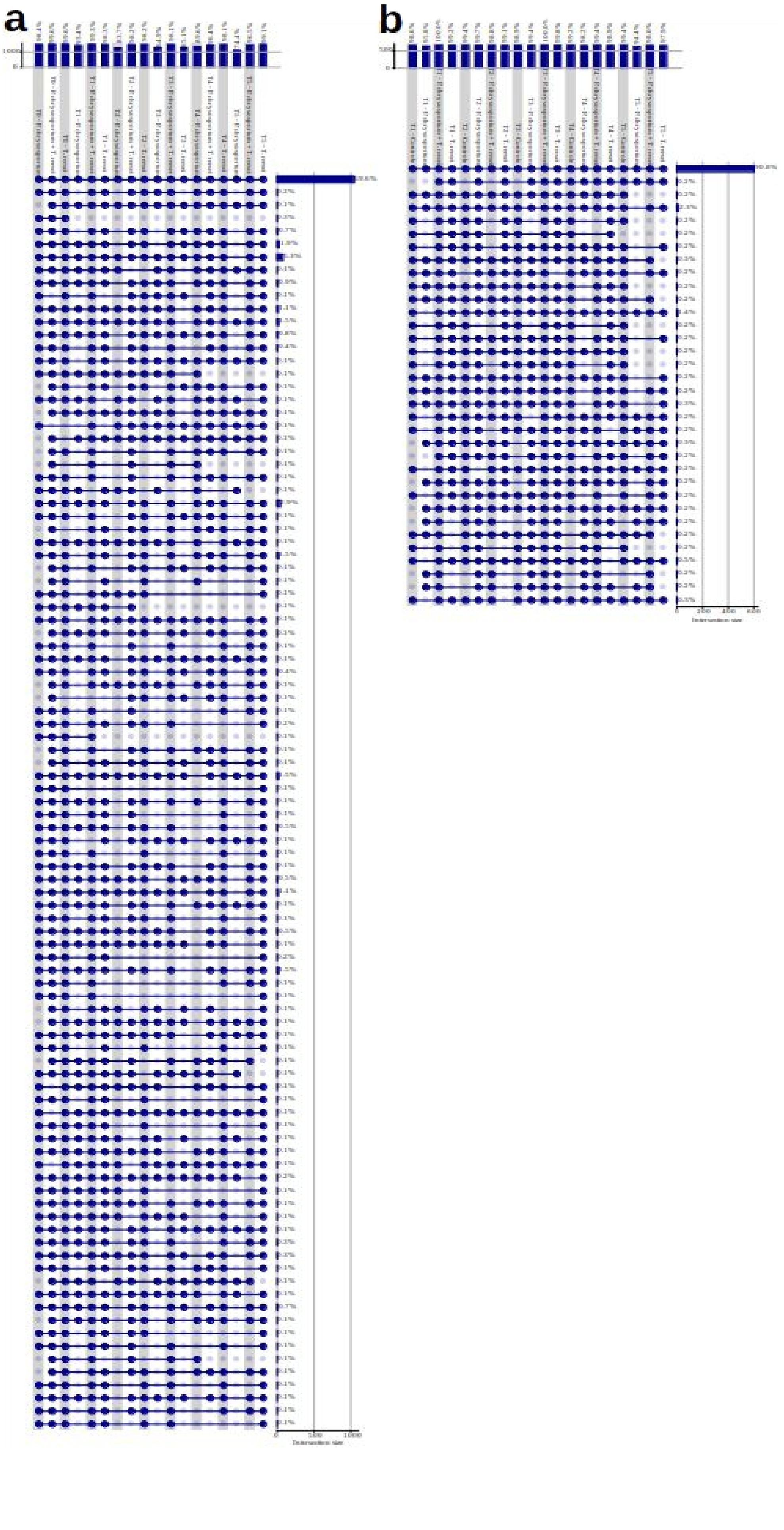

Figure S6. Intersection analysis of the metabolites detected in the different culture conditions (*T. reesei*, *P. crhysosporium* and co-cultivation). Treatments are represented in the columns and intersections are depicted in a row. Percentages represent the amount of metabolites observed for each treatment (columns) or intersections (rows). A) Intersection analysis of the metabolites obtained in the bioreactor cultivation. B) Intersection analysis of the metabolites obtained in the validation experiment.

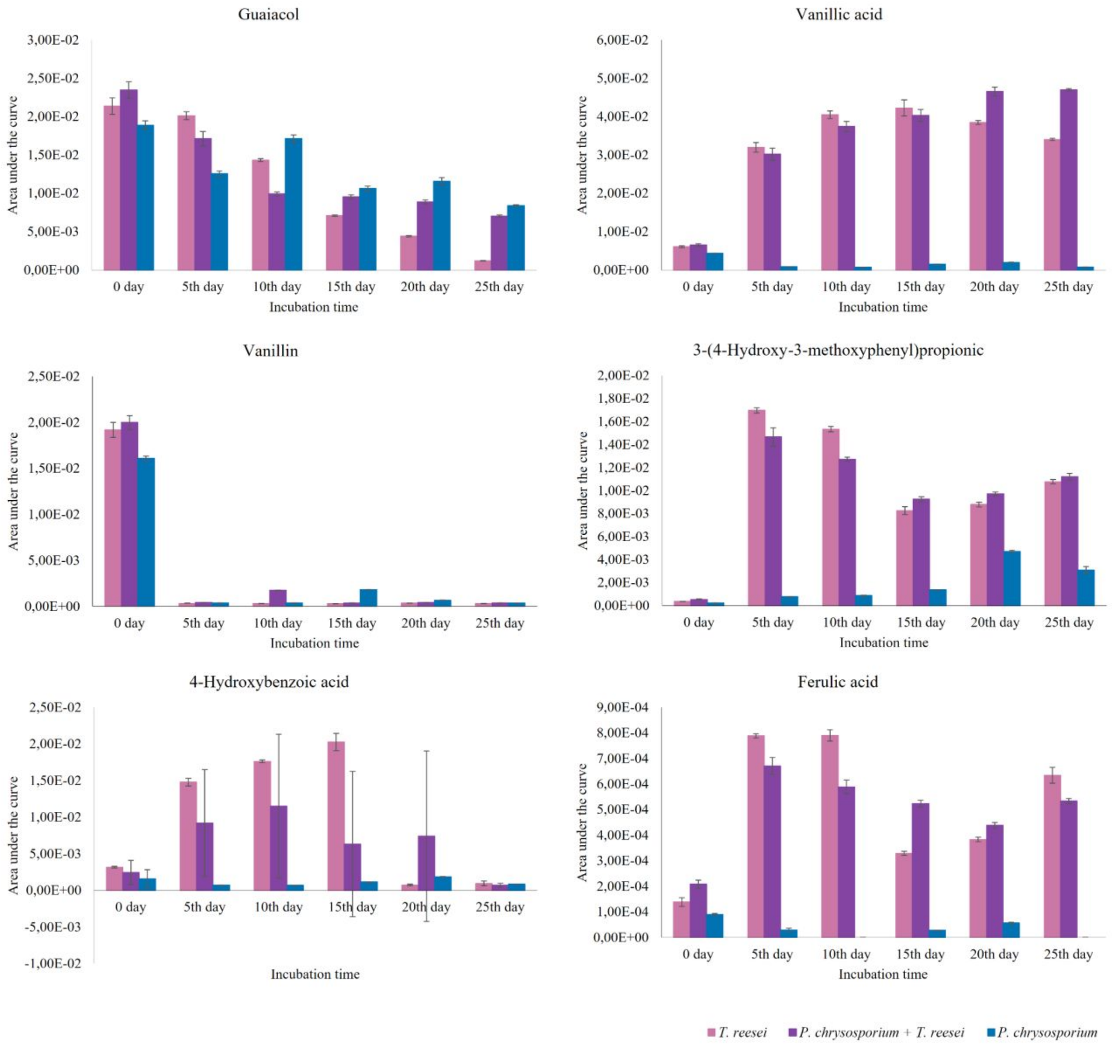


Figura S7. Mean and standard deviation (error bars) for the three technical replicates for lignin metabolism intermediaries' Area under de curve over time.

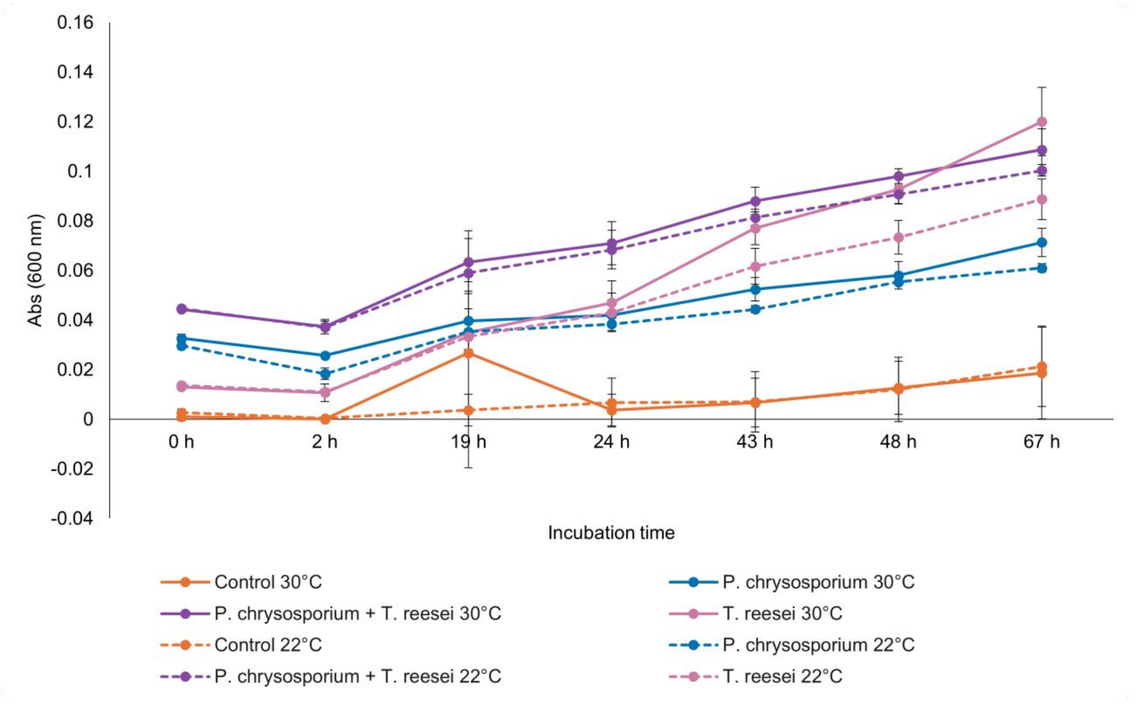


Figure S8. Growth curve on modified Czapek Dox medium for *P. chrysosporium*, *T. reesei*, co-culture, and control. The error bars represent the standard deviation for three biological replicates.

Table S5. Average Absorbance (600 nm) comparison for time 67 h.

| **Comparison** | **Time** | **t_stat** | **p_valor** |
|---|---|---|---|
| P. chrysosporium 30°C x T. reesei 30°C | 67 horas | -5.66589884 | 0.00478486 |
| P. chrysosporium 22°C x T. reesei 22°C | 67 horas | -5.75501454 | 0.00452105 |
| P. chrysosporium 30°C x Co-cultivo 30°C | 67 horas | -6.38179228 | 0.0030934 |
| P. chrysosporium 22°C x Co-cultivo 22°C | 67 horas | -23.6 | 1.91127268e-05 |
| T. reesei 30°C x Co-cultivo 30°C | 67 horas | 1.21895906 | 0.2898278 |
| T. reesei 22°C x Co-cultivo 22°C | 67 horas | -2.38698019 | 0.07541478 |

Table S6. Chlorinated compounds detected in GC-MS.

| Compound_Name | BLANK1 | BLANK2 | BLANK3 | SAMPLE1 | SAMPLE2 | SAMPLE3 |
|---|---|---|---|---|---|---|

| | | | | | | |
|---|---|---|---|---|---|---|
| Succinic acid, 2,4,6-trichlorophenyl 2-naphthylmethyl ester | 9414,596335 | 10947 | 17306 | 3189,606191 | 6309,518284 | 8732,6624 |
| 4-Chloro-2-fluoroaniline | 38920,2 | 61386,9 | 151710,9754 | 21827,89761 | 33738,84941 | 51608,15 |
| Trichloroacetic acid, pentafluorobenzyl ester | 7146,6 | 14110,25 | 30675,25 | 17433,35908 | 9805,146866 | 17800,203 |
| 3-Azahexane, N-[3,4-dichlorophenylacetyl]-1-[pyrrolidin-1-yl]- | 3961,55 | 14825,8 | 21676,5 | 3858,214286 | 10840,21245 | 13314,257 |
| 1H-Indole, 5-chloro-2,3-dihydro-1,3,3-trimethyl-2-methylene- | 145797,3063 | 254650,0247 | 478527,5507 | 74328,58614 | 165533,2889 | 198454,2 |
| "2,4,5-Trichlorophenyl cinnamate" | 10010,1 | 11911,2 | 18629,8 | 2955,835714 | 16861,40381 | 21024,549 |
| 2,4-Dichlorobenzoic acid, TMS derivative | 0 | 0 | 2079 | 740242,9393 | 636688,3052 | 966910,43 |
| (Benzooxazol-2-yl)(2-chlorophenyl)amine | 922652,6303 | 1154234,667 | 1670065,08 | 380023,8642 | 747483,3903 | 414927,0 |
| 2-(6-Chloro-3-nitro-4-phenyl-quinolin-2-ylsulfanyl)-1-(2,3-dihydro-benzo[1,4]dioxin-6-yl)- ethanone | 10946,95 | 174001,0217 | 148886,4 | 11402,88737 | 34599,812 | 70069,386 |
